# Genome Sequence of *Aeromonas hydrophila* Strain AH-3 (Serotype O34)

**DOI:** 10.1128/genomeA.00919-16

**Published:** 2016-09-01

**Authors:** Gabriel Forn-Cuní, Juan M. Tomás, Susana Merino

**Affiliations:** Departamento de Genética, Microbiología y Estadística, Sección Microbiologia, Virología y Biotecnología, Facultad de Biología, Universidad de Barcelona, Barcelona, Spain

## Abstract

*Aeromonas hydrophila* is an emerging pathogen of poikilothermic animals, from fish to mammals, including humans. Here, we report the whole-genome sequence of the *A. hydrophila* AH-3 strain, isolated from a fish farm goldfish septicemia outbreak in Spain, with a characterized polar and lateral flagellum glycosylation pattern.

## GENOME ANNOUNCEMENT

Much evidence in recent years has shown that protein glycosylation, once thought to be a eukaryote-only posttranslational modification, is not only present in bacteria but also plays a key role in bacterial infectivity and virulence (1, 2). Protein glycosylation in bacteria involves mainly surface array proteins and flagellin, although the mechanisms governing this phenomenon are not completely elucidated and appear to be species specific (3).

*Aeromonas hydrophila* AH-3, which was recently classified as *Aeromonas piscicola* AH-3 (4), is linked to gastroenteric infections and has a different and characterized glycosylation pattern in both the polar and lateral flagella (5). Similar to other well-known bacterial virulence factors, flagellar glycosylation on this strain affects both bacterial colonization and the inflammatory response in human cultured cells (3). The analysis of the genome from this strain will allow in-depth understanding of the genes involved in the glycosylation process in bacteria and its importance in virulence and pathogenicity.

The genome of *A. hydrophila* strain AH-3 was fully sequenced using Illumina MiSeq II, generating a total of 5,456,534 paired reads with 84× coverage. Read quality analysis and trimming were done with Prinseq 0.20.4 (6). *De novo* assembly with SPAdes 3.6.0 (7) resulted in 82 scaffolds larger than 500 kb.

Genome annotation was performed both via the NCBI Prokaryotic Genome Annotation Pipeline (PGAAP) and Rapid Annotations using Subsystems Technology (RAST). The complete genome of *A. hydrophila* AH-3 is 4,886,878 bp, with 59.8% G+C content, and codes for 4,399 predicted genes, five rRNAs, and 79 tRNA sequences.

### Accession number(s).

This whole-genome shotgun project has been deposited at DDBJ/EMBL/GenBank under the accession number LYXO00000000 (BioProject PRJNA323710). The version described in this paper is the first version, LYXO01000000.

## References

[1] Benz I, Schmidt MA 2002 Never say never again: protein glycosylation in pathogenic bacteria. Mol Microbiol 45:267–276. doi:10.1046/j.1365-2958.2002.03030.x. 12123443

[2] Tan FY, Tang CM, Exley RM 2015 Sugar coating: bacterial protein glycosylation and host-microbe interactions. Trends Biochem Sci 40:342–350. doi:10.1016/j.tibs.2015.03.016. 25936979

[3] Merino S, Tomás JM 2014 Gram-negative flagella glycosylation. Int J Mol Sci 15:2840–2857. doi:10.3390/ijms15022840. 24557579 PMC3958885

[4] Beaz-Hidalgo R, Alperi A, Figueras MJ, Romalde JL 2009 *Aeromonas piscicola* sp. nov., isolated from diseased fish. Syst Appl Microbiol 32:471–479. doi:10.1016/j.syapm.2009.06.004. 19570633

[5] Wilhelms M, Fulton KM, Twine SM, Tomás JM, Merino S 2012 Differential glycosylation of polar and lateral flagellins in *Aeromonas hydrophila* AH-3. J Biol Chem 287:27851–27862. doi:10.1074/jbc.M112.376525. 22733809 PMC3431630

[6] Schmieder R, Edwards R 2011 Quality control and preprocessing of metagenomic datasets. Bioinformatics 27:863–864. doi:10.1093/bioinformatics/btr026. 21278185 PMC3051327

[7] Bankevich A, Nurk S, Antipov D, Gurevich AA, Dvorkin M, Kulikov AS, Lesin VM, Nikolenko SI, Pham S, Prjibelski AD, Pyshkin AV, Sirotkin AV, Vyahhi N, Tesler G, Alekseyev MA, Pevzner PA 2012 SPAdes: a new genome assembly algorithm and its applications to single-cell sequencing. J Comput Biol 19:455–477. doi:10.1089/cmb.2012.0021. 22506599 PMC3342519

